# Case Report: Successful Immunotherapy Improved the Prognosis of the Unfavorable Subset of Cancer of Unknown Primary

**DOI:** 10.3389/fimmu.2022.900119

**Published:** 2022-06-22

**Authors:** Jie Mei, Hao Wang, Honghong Fan, Junli Ding, Junying Xu

**Affiliations:** Department of Oncology, The Affiliated Wuxi People’s Hospital of Nanjing Medical University, Wuxi, China

**Keywords:** cancers of unknown primary, immunotherapy, PD-L1, molecular profiling, combinational therapy, case report

## Abstract

**Background:**

Cancer of unknown primary (CUP) is heterogeneous and has a wide variety of clinical presentations and a poor prognosis in most patients, with a median overall survival of only 6 months. The development of molecular profiling contributes to precision therapy, and targeted drugs and immune checkpoint inhibitors (ICIs) greatly promote individualized treatment.

**Case presentation:**

Here, we reported a case of an unfavorable subset of CUP who had a long time of survival after the immunotherapy-prominent comprehensive treatment. A 48-year-old man presented with back pain and a cough. A diagnostic work-up showed bone marrow, multiple bones, and lymph node metastasis. Lymph node pathology implies metastatic poorly differentiated cancer. Next-generation sequencing (NGS) showed no special targets, but the tumor proportion score (TPS) of programmed death-ligand 1 (PD-L1) was 80% and the tumor mutation burden (TMB) was 16.7 per million bases. After two cycles of pembrolizumab 200 mg D1 plus nanoparticle albumin-bound (nab)-paclitaxel 200 mg D1&8 (q3w), PET-CT and bone marrow aspiration cytology showed a complete response (CR). Subsequently, pembrolizumab alone was used for three months. The left inguinal lymph nodes showed new metastasis. After two cycles of the combination treatment of pembrolizumab and (nab)-paclitaxel, a partial response (PR) was achieved. After seven months, retroperitoneal lymph nodes showed new metastasis, and the sequential treatment with radiotherapy and pembrolizumab exhibited encouraging efficacy. To date, the patient has survived nearly 40 months with the combination therapy.

**Conclusions:**

The ICI-prominent comprehensive treatment provided clinical benefit for the reported case of CUP. Thus, CUP patients with markers of benefiting from immunotherapy should be actively treated with immunotherapy to improve their prognosis.

## Introduction

Cancer of unknown primary (CUP) is defined as a histologically confirmed metastatic tumor whose primary site cannot be identified during standard pretreatment evaluation (1). These heterogeneous tumors account for 2 to 5% of all diagnosed cancers and have a wide variety of clinical presentations (2). Early dissemination, aggressiveness, and unpredictability of metastatic patterns are characteristic of these tumors (3). Given this background, the treatment of CUP is problematic and has not been well developed. More than 80% of patients with CUP have a poor prognosis with a median survival of only 6 months (4). However, patients diagnosed with CUP with colorectal, lung, and renal profiles seem to belong to the good prognostic subsets of CUP. Based on the available data, the treatment of these patients, similar to the correspondent primary tumors, seems promising. However, the absence of defining/validated diagnostic criteria for CUP with lung and renal profiles limits the interpretation of the treatment outcomes (5).

Here, we report a CUP patient who fell into an unfavorable subset with bone marrow metastasis and multiple bone and lymph node metastases, who has survived nearly 40 months to date. The treatment plans were made according to molecular profiling and immunohistochemistry (IHC) analysis. The immune checkpoint inhibitor (ICI)-prominent comprehensive treatment conferred a survival benefit on the patient.

## Case Presentation

**Figure 1:**
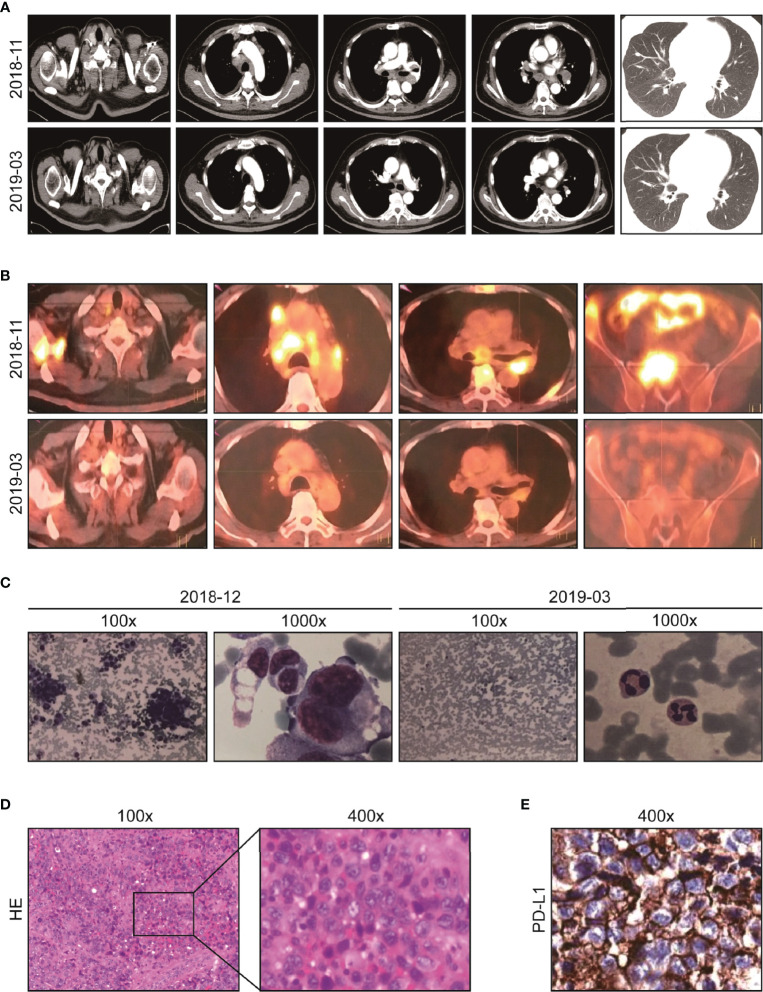
Imaging and pathological data of the patient at diagnosis and after initial immuno-chemotherapy. **(A)** Representative chest CT before (upper) and after treatment (lower): abnormal lymph node in supraclavicular and mediastinum disappeared after two cycles of immuno-chemotherapy. **(B)** Representative PET-CT before (upper) and after treatment (lower): lesions with increased FDG uptake completely disappeared, and new bone generated after two cycles of immuno-chemotherapy. **(C)** Representative images of bone marrow aspiration cell smear: diffusely proliferative malignant tumor cells completely disappeared after two cycles of immuno-chemotherapy. **(D)** Pathological analysis (HE staining) of resected left supraclavicular lumps. **(E)** The protein expression of PD-L1 analyzed by IHC test (antibody clone number: 22C3).

On 29 October 2018, a 48-year-old man sought medical advice because of back pain and a cough for forty days. He had been examined in the Department of Respiration at our hospital and Shanghai Tumor Hospital, but the diagnosis was unclear. A physical examination exhibited an enlarged lymph node palpable on the left supraclavicular with tenacity in texture without tenderness, and other physical examinations showed no obvious abnormalities. His Eastern Cooperative Oncology Group performance status (ECOG-PS) score was 2. Chest CT showed multiple enlarged lymph nodes in the mediastinum with some nodules in both lung fields (**Figure 1A**, upper). PET-CT showed that FDG uptake significantly increased in multiple enlarged lymph nodes (bilateral clavicle area, left armpit, and mediastinum) and multiple bones (thoracic spine, lumbar spine, sacral spine, pelvis, scapula, sternum, and ribs), implying extensive lymph node metastases and bone destruction (**Figure 1B**, upper). On 28 November 2018, the biopsy of the right scapula soft tissue under ultrasound guidance was performed at the Shanghai Tumor Hospital. Unfortunately, only skeletal muscle, fibrous tissue, and individual epithelioid cells were shown. On 19 December 2018, the patient was admitted to the Department of Oncology. Bone marrow aspiration cell smear showed scattered and clustered metastatic cancer cells. A bone marrow biopsy showed diffusely proliferative malignant tumor cells of epithelial origin (**Figure 1C**, left). Some enlarged lymph nodes are touched by the bilateral clavicle through physical examination. A left supraclavicular lymphadenectomy biopsy and a bone marrow biopsy were performed. Lymph node pathology implied metastatic poorly differentiated cancer (**Figure 1D**) and a large series of IHC markers (AE1/AE3+, EMA+, CK7−, CK5/6−, CK20−, HMB45−, S-100−, CD138−, CD38−, CD20−, CD3−, P40−, NapsinA−, TTF1−, PAX8−, CD30−, EBER−, CDX2−, Syn−, CgA−, CD56−, Ki-67: 80%+) were unable to identify the tissue source. Additionally, the primary site could not be indicated in spite of consultation at Shanghai Tumor Hospital as well. Then, next-generation sequencing (NGS) of the lymph node tissue and peripheral blood was adopted. No special targets were found (**Table 1**). Fortunately, the tumor proportion score (TPS) of programmed death-ligand 1 (PD-L1) (**Figure 1E**) and tumor mutation burden (TMB) was 16.7 per million bases. The patient never smoked or drank alcohol and had no family history of cancer. Additionally, the basic thyroid function of the patient, myocardial zymogram, brain natriuretic peptide, cortisol, adrenocorticotropic hormone, and sex hormone were within normal limits, except for type 2 diabetes and chronic hepatitis B. Insulin and entecavir were used to control blood sugar and virus replication in the normal range.

**Table 1:** Summary of gene mutations concluded from NGS analysis.

Gene	Variation type	cDNA variation	Abundance
ARID1A	Alternative splicing	c.3198+1G>T	7.04%
ARID1A	Missense	c.1928C>G	4.96%
ARID2	Nonsense	c.2666C>G	21.38%
ARID2	Nonsense	c.1991C>G	5.95%
DNMT3A	Missense	c.1222G>A	11.26%
FANCA	Rearrangement	LINC01572-FANCA	33.79%
GALNT12	Missense	c.845G>A	12.23%
HIST1H2BD	Missense	c.41G>T	12.81%
IKZF1	Missense	c.154G>T	6.94%
KIT	Amplification	–	CN3.56
KIT	Rearrangement	EPHA5-KIT	37.78%
LRP1B	Missense	c.9793C>T	7.32%
NF1	Alternative splicing	c.6757-1G>A	15.01%
NOTCH3	Missense	c.1136G>C	18.07%
PDGFRA	Amplification	–	CN4.89
RB1	Missense	c.1183C>G	12.11%
ROS1	Missense	c.4906C>T	15.03%
TERT	Missense	c.3376G>T	3.97%
TP53	Missense	c.733G>T	11.78%

On 29 December 2018, “docetaxel 140 mg D1 plus carboplatin 600 mg D2 (q3w)” chemotherapy regimens were adopted, which resulted in grade four myelosuppression. The patient recovered after treatment with granulocyte colony-stimulating factor and a transfusion of red blood cells and platelets. Therefore, chemotherapy was difficult to implement. Given the PD-L1 TPS was 80%, pembrolizumab 200 mg D1 (q3w) was applied on 11 January 2019. After only one pembrolizumab treatment, the physical strength of the patient improved, and the pain in the back and waist decreased significantly. Morphine was reduced from 60 mg q12 h to 30 mg q12 h. According to the coordination of chemotherapy and immunotherapy, nanoparticle albumin-bound (nab)-paclitaxel, free of dexamethasone, pretreatment was adopted. Then, two cycles of pembrolizumab 200 mg D1 plus nab-paclitaxel, 200 mg D1&8 (q3w) were carried out. Fortunately, the ECOG-PS score was 0 and morphine was discontinued. Chest CT was used and showed no obvious lesions (**Figure 1A**, lower). In order to fully evaluate the therapeutic effect, PET-CT was used again after the agreement of the patient. Amazingly, the original FDG metabolism increased focus completely disappeared and enlarged lymph nodes disappeared (**Figure 1B**, lower). A bone marrow biopsy showed no proliferative malignant tumor cells (**Figure 1C**, right). The efficacy evaluation was complete response (CR), and pembrolizumab 200 mg D1 (q3w) was used as maintenance therapy.

**Figure 2:**
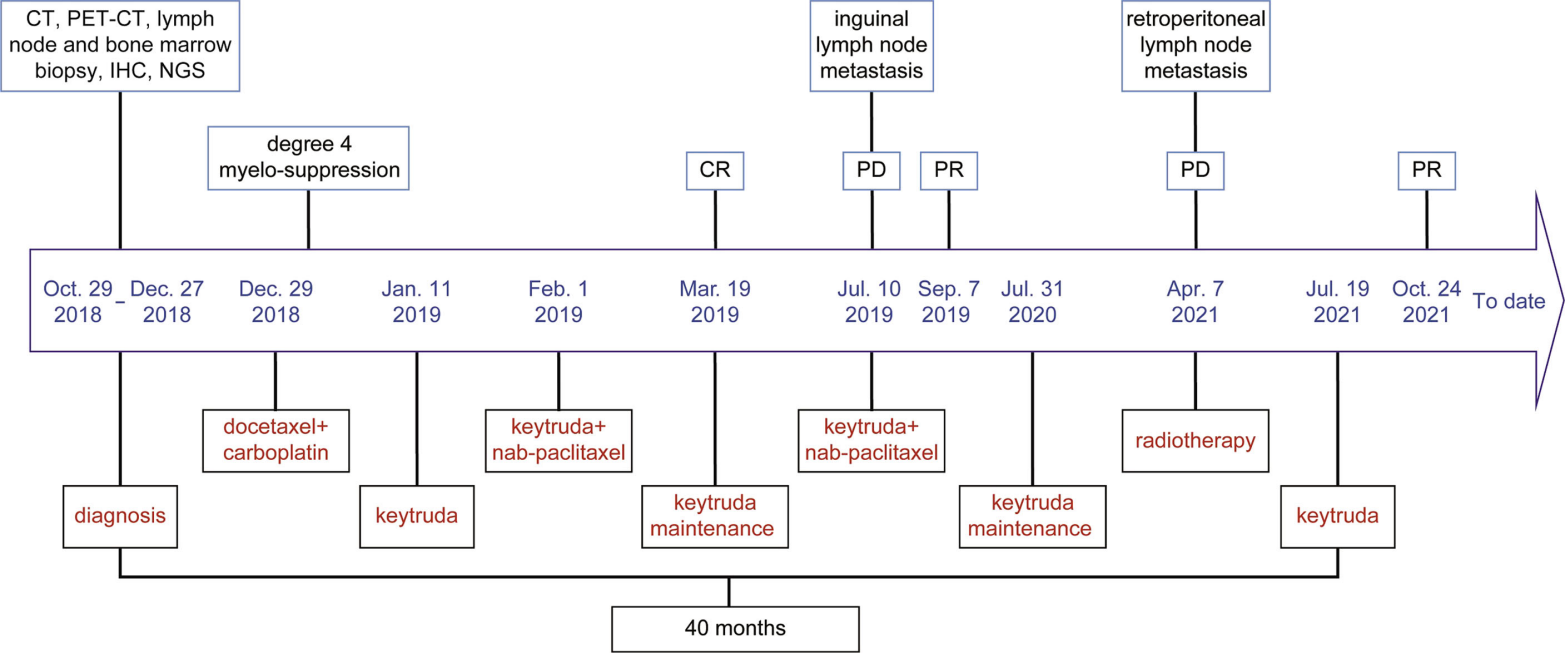
The timeline of the treatment process for the patient. The patient has experienced immunotherapy, the combination of immunotherapy and chemotherapy, and the combination of immunotherapy and radiotherapy. At initial treatment and after two recurrences, the patient achieved clinical benefits.

**Figure 3:**
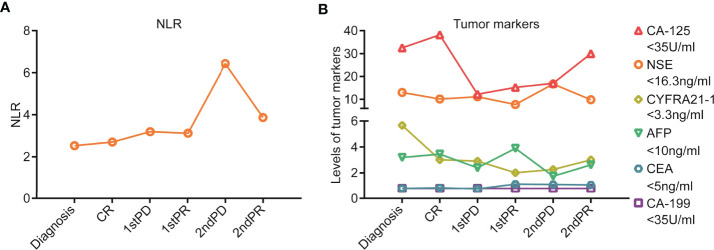
Blood test results during the treatment process for the patient. **(A)** NLR value and its dynamic changes during the treatment process. **(B)** Tumor marker levels and their dynamic changes during the treatment process, including CEA, AFP, CA199, CA125, NSE, and CYFRA21-1.

After three cycles of pembrolizumab 200 mg D1 (q3w) treatment, on 8 July 2019, the patient found lumps in the left inguinal. A color doppler ultrasound showed an enlarged lymph node (**Figure S1A**) and a biopsy with a coarse needle was applied. The pathological result was still metastatic poorly differentiated carcinoma (**Figure S1B**). Meanwhile, enhanced CT of the chest and abdomen and spinal MRI showed no relapse. At this point, the patient was diagnosed with progressive disease (PD). Then, from 10 July 2019, pembrolizumab 200 mg D1 plus nab-paclitaxel 200 mg D1&8 (q3w) were re-applied. It was encouraging to see that the enlarged lymph nodes in the left inguinal were reduced after two cycles, reaching a partial response (PR), and the ECOG-PS score was still 0. The efficacy was reconfirmed four weeks later. From 31 July 2020, pembrolizumab 200 mg D1 (q3w) was used as maintenance therapy. However, on 5 April 2021, retroperitoneal lymph nodes showed new metastasis, and the sequential treatment with radiotherapy (dose unknown outside our hospital) and pembrolizumab 200 mg D1 (q3w) exhibited encouraging efficacy, and efficacy evaluation reached PR (**Figure S2**). Then, pembrolizumab 200 mg D1 (q3w) was used as maintenance therapy. To date (1 March 2022), the patient has survived nearly 40 months with the combination therapy. The treatment process is shown in **Figure 2**. Additionally, we also summarized the changes of several significant blood test results, including NLR and tumor markers (CEA, AFP, CA199, CA125, NSE, and CYFRA21-1), but no significant clinical importance was observed (**Figure 3**). The patient chose outpatient treatment in most cases and worked for the company.

## Discussion and Conclusions

CUP is heterogeneous and has a wide variety of clinical presentations and a poor prognosis in most patients (6). As for treatment, it is paramount to identify patients who fall into one of the defined “favorable” (20% of CUP) or “unfavorable” subset categories (7). The percentage of unfavorable patients who do not fit into the previously historically defined favorable subsets is substantially smaller than several years ago, and the favorable subset group is becoming larger (8). Therefore, the diagnosis is very significant. A diagnosis includes a thorough patient history, gender, clinical examination, a basic lab draw with the most relevant tumor markers, and contrast-enhanced chest/abdominal/pelvic CT. PET/CT may be warranted in some situations, especially when considering local or regional therapy or in the cervical lymph nodes suggestive of head and neck cancer (9, 10). IHC analysis plays an important role in the diagnostic work-up in CUP, characterization of the tumor type. Nowadays, molecular techniques including mutational testing with next-generation sequencing and tissue of origin predicting with gene expression profiling with a 92-gene assay have strongly aided diagnostics (11, 12). Additionally, the EPICUP and other platforms based on gene or microRNA expression have begun to be implemented, in conjunction with current diagnostic strategies, in routine clinical practice. The application of these diagnostic tools in clinical settings represents an advance in the classification of CUP. The prediction of the tissue of origin can prompt the further interrogation of particular genetic alterations for which specific treatments exist (13).

The current patient fell into the unfavorable subset category because of bone marrow metastatic carcinoma, severe pain, an ECOG-PS score of 2, multiple lymph nodes, and bone metastases. The primary site could not be indicated on the basis of multiple IHC results or even with the consultation at Shanghai Tumor Hospital, the authoritative pathology consultation center. The use of IHC in CUP is based on the premise that concordance exists in the expression profiles of primary and metastatic cancers. However, limitations of IHC testing include factors affecting tissue antigenicity, interobserver and intraobserver variability in interpretation, tissue heterogeneity, and inadequate biopsy samples. IHC studies are useful for the characterization of CUP tumors by providing information about tumor lineage, cell type, and pathological diagnosis. However, exhaustive IHC studies (over 10–12 stains) have not been shown to increase the diagnostic accuracy in identifying the putative primary sites (14). For the current patient, the diagnosis was tortuous and took up to two months. Therefore, testing for a large series of IHC markers in individual patients should be avoided.

Over the past decade, gene expression profiling (GEP) assays in CUP were designed to identify the tissue of origin. When validated using samples from known tumor types, these assays have generally demonstrated an accuracy rate of 85 to 90% (15). However, because it is difficult to confirm the site of origin in most cases of CUP, the accuracy of GEP assays in occult primary tumor samples is challenging to determine (14). Another active area of investigation has been the use of NGS to characterize the genome of CUP. NGS can identify potentially actionable biomarkers outside tissue-specific markers. For example, mutations and/or amplification of ERBB2, EGFR, and BRAF occur more frequently in adenocarcinoma CUPs (10, 8, and 6%, respectively) than in non-adenocarcinoma CUPs (4, 3, and 4%, respectively) (16). In a recent comprehensive study of 200 CUP specimens, the use of a hybrid-capture-based NGS assay enabled the identification of at least 1 potentially targetable genomic alteration in 85% of the CUP specimens (12). But the clinical benefit of the targeted treatment of CUP based on molecular studies remains controversial (17). Thus, pathologists and oncologists must collaborate on the judicious use of these modalities on a case-by-case basis with the best possible individualized patient outcome in mind.

For our patient, before the NGS assay result was known, palliative treatment with empirical chemotherapy was adopted, but severe myelosuppression occurred because of bone marrow metastasis. Fortunately, the high expression of PD-L1 implied the efficacy of immunotherapy. For advanced cancer therapy, a dramatic improvement in survival has been achieved with ICIs (18). Various malignancies, including non-small cell lung cancer, gastroesophageal cancer, genitourinary cancer, and head and neck cancer, postmortem analysis and gene expression profiling have identified these cancer types as common occult origins of CUP (19), suggesting that ICIs also prove effective for treating CUP. Haratani et al. have proved that the immune profiling of CUP is similar to that of malignancies responsive to ICIs (20), and thus ICIs might be a potential option for CUP treatment. Additionally, ICIs seem to be the most reasonable approach for patients with melanoma of unknown primary (MUP), considering the vigorous immune anti-melanoma response at baseline. However, BRAF/MEK inhibitors also appear to induce immune responses by affecting the tumor microenvironment and immune surveillance. MUP patients on immunotherapy probably display better outcomes compared to those with melanoma of the known primary subset (21). For our patient, who had only once used pembrolizumab, the limb pain was obviously relieved. Morphine was reduced by half. Additionally, the present patient harbored an NF1 mutation, which has been indicated as a favorable biomarker for anti-PD-1 immunotherapy (22). Indeed, several trials evaluating the efficacy of ICIs in CUP patients are currently in progress, including phase II trials of pembrolizumab (NCT03391973 and NCT03752333). Additionally, the open-label phase II trial on the efficacy of nivolumab in CUP has been reported and demonstrates a clinical benefit of nivolumab for patients with CUP (23).

ICI combination strategies with curative potential have been focused on in the field of cancer therapy. Antiangiogenesis agents, cytotoxic chemotherapeutic drugs, and radiotherapy are the most commonly used combination therapies (24). The disseminated disease in the patient required chemotherapy. Paclitaxel is the base regimen for CUP, but high-dose dexamethasone pretreatment is prohibited in ICI therapy. More importantly, Nab-paclitaxel, free of dexamethasone, more importantly, has significantly improved overall survival in several refractory malignancies as the first-line treatment, such as pancreatic cancer, triple-negative breast cancer, and advanced non-small-cell lung cancer (25–27). The high efficiency and low toxicity of nab-Paclitaxel might take advantage of enhanced concentration of free taxon, active transportation through endothelial cells, and penetration into tumor tissue by albumin binding to secreted protein acidic and rich in cysteine receptor. A recent study has shown that SPARC plays an important role in tumor uptake of nab-paclitaxel (28). Furthermore, a phase 3 trial showed that nab-paclitaxel may enhance the anticancer activity of atezolizumab, a PD-L1 antibody, in untreated metastatic triple-negative breast cancer (29). Therefore, nab-paclitaxel might be a good combination drug with immunotherapy. Additionally, radiotherapy could positively modulate thelandscape of the immune microenvironment and systemic response and enhance the anticancer activity of immunotherapy (30). For this patient, the sequential treatment with radiotherapy and pembrolizumab successfully repressed the progression of retroperitoneal lymph node metastases.

In conclusion, the pembrolizumab-prominent comprehensive treatment provided clinical benefits for the reported case of CUP. Molecular profiling and IHC analysis provided a precise, individualized guide. Overall, we believe that CUP patients with markers of benefiting from immunotherapy should be actively treated with immunotherapy to improve their prognosis.

## Data Availability Statement

The original contributions presented in the study are included in the article/**Supplementary Material**. Further inquiries can be directed to the corresponding authors.

## Ethics Statement

The studies involving human participants were reviewed and approved by the Clinical Research Ethics Committee, The Affiliated Wuxi People’s Hospital of Nanjing Medical University. The patients/participants provided their written informed consent to participate in this study. Written informed consent was obtained from the individual(s) for the publication of any potentially identifiable images or data included in this article.

## Author Contributions

JX, JD, and JM contributed to conception and design. JM and HF performed clinical investigations and interpretation of results. HW, JD, and JX were the clinicians in charge of patient care and management. JM and HF wrote the initial manuscript draft. JX and JD reviewed the manuscript and contributed to the final draft. All authors listed have made a substantial, direct, and intellectual contribution to the work and approved it for publication.

## Funding

This work was supported by the Wuxi Municipal Health Commission Fund (No. M202108) and the Wuxi Science and Technology Bureau Fund (No. N20202018).

## Conflict of Interest

The NGS and IHC analyses were performed by Burning Rock Biotech.

The remaining authors declare that the research was conducted in the absence of any commercial or financial relationships that could be construed as a potential conflict of interest.

## Publisher’s Note

All claims expressed in this article are solely those of the authors and do not necessarily represent those of their affiliated organizations, or those of the publisher, the editors and the reviewers. Any product that may be evaluated in this article, or claim that may be made by its manufacturer, is not guaranteed or endorsed by the publisher.
