# Supplementary material for: Case Report: Successful Immunotherapy Improved the Prognosis of the Unfavorable Subset of Cancer of Unknown Primary
Source: Front Immunol. 2022 Jun 22;13:900119. doi: 10.3389/fimmu.2022.900119 (PMC9256999; doi:10.3389/fimmu.2022.900119)
Supplement: Supplementary file 1 [file DataSheet_1.docx]

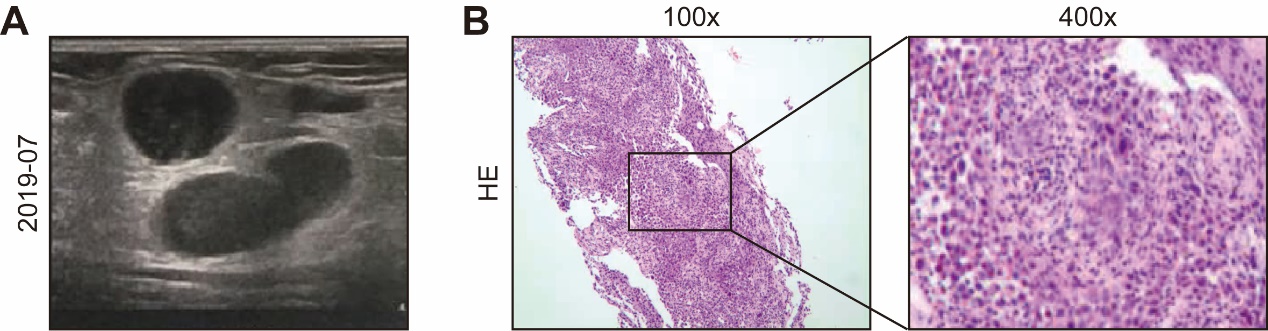


**Figure S1. Imaging and pathological data of the patient at the first progression.**

(A) Ultrasound image of inguinal metastatic lymph nodes: round enlarged lymph nodes with uneven echo and lymphatic hilum disappearance. (B) Pathological analysis (HE staining) of puncture acquired left inguinal lumps.


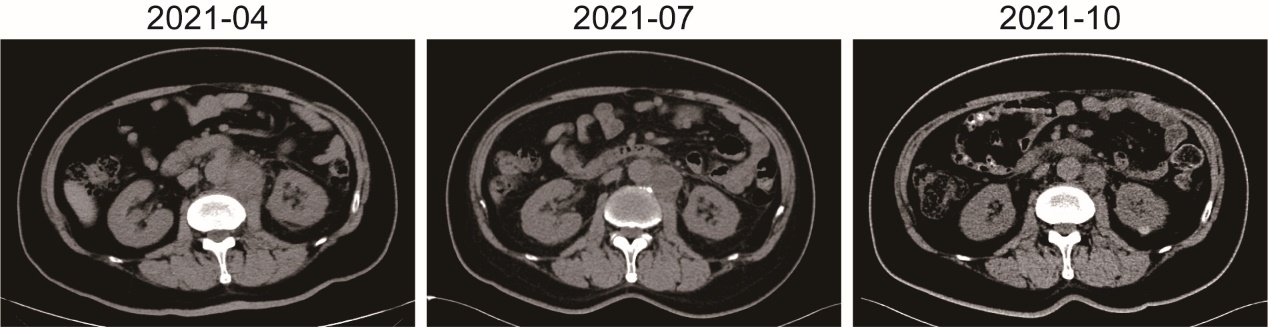


**Figure S2. Imaging data of the patient after radiotherapy and immunotherapy.**

After the sequential treatment of radiotherapy and immunotherapy, the abnormal retroperitoneal lymph node significantly decreased.
